# Impact of physical activity on all-cause mortality in individuals with non-cystic fibrosis bronchiectasis

**DOI:** 10.3389/fmed.2025.1479638

**Published:** 2025-02-26

**Authors:** Sang Hyuk Kim, Hayoung Choi, Kyungdo Han, Jin-Hyung Jung, Bumhee Yang, Hyun Lee

**Affiliations:** ^1^Division of Pulmonary, Allergy, and Critical Care Medicine, Department of Internal Medicine, Korea University Guro Hospital, Korea University College of Medicine, Seoul, Republic of Korea; ^2^Division of Pulmonary, Allergy, and Critical Care Medicine, Department of Internal Medicine, Dongguk University Gyeongju Hospital, Dongguk University College of Medicine, Gyeongju, Republic of Korea; ^3^Division of Pulmonary, Allergy, and Critical Care Medicine, Department of Internal Medicine, Hallym University Kangnam Sacred Heart Hospital, Hallym University College of Medicine, Seoul, Republic of Korea; ^4^Department of Statistics and Actuarial Science, Soongsil University, Seoul, Republic of Korea; ^5^Samsung Biomedical Research Institute, Sungkyunkwan University School of Medicine, Suwon, Republic of Korea; ^6^Division of Pulmonary and Critical Care Medicine, Department of Internal Medicine, Chungbuk National University Hospital, Chungbuk National University College of Medicine, Cheongju, Republic of Korea; ^7^Division of Pulmonary Medicine and Allergy, Department of Internal Medicine, Hanyang University College of Medicine, Seoul, Republic of Korea

**Keywords:** bronchiectasis, mortality, exercise, movement, running, sedentary behavior

## Abstract

**Background:**

Little information is available regarding whether active physical activity lowers mortality risk in individuals with bronchiectasis.

**Methods:**

We used the Korean National Health Insurance Service database from 2010 to 2016 to evaluate the association between changes in physical activity and mortality risk in individuals with bronchiectasis. Of 552,510 individuals with newly diagnosed bronchiectasis, we enrolled 165,842 individuals who had two consecutive health examinations before and after bronchiectasis diagnosis, within two years, as the study aimed to measure changes in exercise habits between the two time points. Active physical activity was defined as engaging in moderate- or vigorous-intensity physical activity at least once a week, either before or after bronchiectasis diagnosis. The outcome measure was all-cause mortality.

**Results:**

During a mean follow-up of 6.2 ± 2.1 years, 10,535 (6.4%) individuals with bronchiectasis died. Individuals with bronchiectasis who were physically active exhibited a lower mortality rate than those who were physically inactive. Mortality reduction was particularly evident in the exercise maintainers group (aHR [adjusted hazard ratio] = 0.69, 95% confidence interval [CI] = 0.64–0.74) and individuals with physical activity ≥1,000 metabolic equivalent of task-min per week (aHR = 0.73, 95% CI = 0.70–0.77) compared to those who were physically inactive.

**Conclusion:**

Engaging in active physical activity is associated with a decreased risk of mortality in individuals with bronchiectasis.

## Introduction

Bronchiectasis is a chronic respiratory disease characterized by abnormal radiological dilation of the bronchi, with respiratory symptoms including chronic cough, purulent sputum, and recurrent chest infections (1). Bronchiectasis-related disease burden is substantial and contributes to increased mortality rates (2, 3). Considering that increased mortality is largely driven by comorbidities such as cardiovascular diseases (3, 4), individuals with bronchiectasis may achieve survival gain through lifestyle modifications, such as exercise intervention. However, this association has not been studied.

Individuals with chronic respiratory diseases, including bronchiectasis, tend to have sedentary lifestyles linked to poorer long-term outcomes (5–7). Appropriate physical activity is recommended for this population, and exercise interventions have shown improvements in exercise capacity, dyspnea, and quality of life (8). Furthermore, enhanced physical activity may prevent the development of cardiovascular disease in individuals with bronchiectasis (4). Hence, our study aimed to assess whether physical activity could reduce the mortality risk in individuals with bronchiectasis.

## Methods

### Data source and study population

We used data obtained from the Korean National Health Insurance Service (NHIS) database, which encompasses a large-scale nationwide population-based cohort study (9). The NHIS offers detailed reports that include data from health screening exams, sociodemographic data, self-reported survey responses, clinical laboratory results, information on inpatient and outpatient services, prescription details, and diagnoses classified under the International Classification of Diseases, 10th Revision (ICD-10) codes.

During study participant enrollment, the Korean government mandated general health examinations for health insurance subscribers aged ≥40 years, local subscribers aged ≥20 years, job subscribers of all ages, and Medicaid beneficiaries aged 19–64 years, conducted annually or every 2 years. The health examination included various screening items, such as anthropometric measurements (e.g., body mass index [BMI], waist circumference, and blood pressure), laboratory test results, and questionnaires covering smoking habits, alcohol intake, and physical activity. More detailed information on this database is presented in previous studies (4, 10, 11).

Of 552,510 individuals with newly diagnosed bronchiectasis between January 1, 2010, and December 31, 2016, we enrolled 165,842 individuals, meeting the following criteria: (1) aged over 20 years, (2) ICD-10 codes for bronchiectasis (J47, except E84 [cystic fibrosis]), (3) had two consecutive health examinations before and after bronchiectasis diagnosis, within 2 years, as the study aimed to measure changes in exercise habits between the two time points, (4) no history of prior myocardial infarction (I21–I22) (12, 13) or stroke (I63–I64) (14), as cardiovascular diseases are significant causes of mortality in individuals with chronic respiratory diseases and also influence physical activity levels, and (5) no missing data.

### Exposure: physical activity

The intensity and frequency of physical activity were evaluated to assess exercise habits through the Korean version of the International Physical Activity Questionnaire (IPAQ) (15), a self-reported survey administered during two health screening examinations conducted before and after the bronchiectasis diagnosis. The IPAQ has been broadly used in several high-quality studies (16, 17). The questionnaire contains the amount of time participants typically spend engaging in various physical activities during a typical week using the 7-day recall method. The survey included three questions addressing the frequency and intensity of the following activities: (1) vigorous physical activity lasting at least 20 min, (2) moderate physical activity lasting at least 30 min, and (3) light physical activity lasting at least 30 min. Vigorous-intensity physical activity is an exercise causing significant shortness of breath, such as running or cycling at high speed. Moderate-intensity physical activity is an exercise inducing mild shortness of breath, including brisk walking or cycling at a regular pace. Light physical activity is walking at a slow or leisurely pace.

We calculated the total metabolic equivalent of task (MET)-minutes per week by summing the standardized intensity levels. Using the Ainsworth et al. compendium, an average MET score was derived for each type of activity: 3.3 METs for walking, 4.0 METs for moderate-intensity activities, and 7.0 METs for vigorous-intensity activities (18). This approach demonstrated validity and reliability in the Korean population when compared to directly measured METs using an omnidirectional accelerometer (19).

Individuals were categorized as either active or inactive, based on their reported physical activity levels. Individuals who reported moderate- or vigorous-intensity physical activity at least once a week, either before or after diagnosis, were categorized as having active activity; individuals who did not meet this criterion were categorized as having inactive activity (20). Active physical activity was further divided into <500, 500–999, and ≥ 1,000 MET-min/week based on the recommendations for physical activity, which has also been widely used for the Korean population (7, 21–23).

Among individuals who engage in active physical activity, changes in exercise habits were assessed and classified into three groups: new exercisers (having inactive physical activity before diagnosis but engaging active physical activity after diagnosis), exercise discontinued (engaging active physical activity before diagnosis changed to having inactive physical activity after diagnosis), and exercise maintainers (engaging active physical activity before and after diagnosis).

### Outcome

The outcome was all-cause mortality, excluding accidental deaths, and data were obtained from the National Death Registry (3, 24–30). Individuals were followed up until either the date of death or the last follow-up date (December 31, 2020), depending on whichever occurred earlier.

### Covariates

Demographics, anthropometric measurements, personal habits, and socioeconomic status were evaluated using previous health examination data close to the time of bronchiectasis diagnosis, and comorbidities were identified within 1 year preceding the diagnosis. Low income was classified as being in the lowest 20% of income distribution within the Korean population or receiving medical aid (31). Smoking status and alcohol use were determined through a self-reported questionnaire (32). Heavy alcohol consumption was defined as an intake of ≥30 g of alcohol per day (33). Comorbidities were identified using ICD-10 codes, supplemented by specific measurements or medication usage when applicable (34–44).

### Statistical analyses

Data are expressed as means with standard deviations or numbers (percentages), as appropriate. Differences in baseline characteristics were assessed using the *t*-test or χ^2^ test, as appropriate. The mortality rate was calculated by dividing the number of deaths by the total follow-up duration (per 1,000 person-years). Cox proportional hazards regression analyses were used to evaluate mortality risk based on the levels of physical activity. The multivariate model was adjusted for age, sex, low income, smoking status, alcohol consumption, BMI, and comorbidities (hypertension, diabetes mellitus, dyslipidemia, asthma or chronic obstructive pulmonary disease [COPD], and cancer). Proportional hazard assumptions were evaluated using graphical methods. Kaplan–Meier curves depicting the probability of mortality were plotted based on changes in exercise habits. All analyses were performed using SAS 9.3 (SAS Institute, Cary, NC, United States). All tests were two-tailed, and *p*-values <0.05 were considered statistically significant.

## Results

### Baseline characteristics

Baseline characteristics of the study population are shown in Table 1. Of the 165,842 individuals, the majority were aged 40–64 years and 51.6% were women. Individuals with active physical activity were more likely to be old (36.9% vs. 34.7% for ≥65 years, *p* < 0.001), be male (53.5% vs. 45.9%, *p* < 0.001), have a higher BMI (mean, 23.7 vs. 23.5 kg/m^2^, *p* < 0.001), and a higher possibility of belonging to the low-income group (18.3% vs. 15.6%, *p* < 0.001) than those who are physically inactive. Physically active individuals were less likely to be current or past smokers compared to those who are physically inactive (12.7% vs. 15.6%, *p* < 0.001).

**Table 1:** Baseline characteristics of the study population.

Variables	Total (*N* = 165,842)	Inactive physical activity (*n* = 110,499)	Active physical activity (*n* = 55,343)	*p*-value
Age, years				
<40 years, *n* (%)	9,621 (5.8)	7,215 (6.4)	2,406 (4.4)	< 0.001
40–64 years, *n* (%)	97,408 (58.7)	64,907 (58.7)	32,501 (58.7)	
≥65 years, *n* (%)	58,813 (35.5)	38,377 (34.7)	20,436 (36.9)	
Sex				< 0.001
Male, *n* (%)	80,336 (48.4)	50,714 (45.9)	29,622 (53.5)	
Female, *n* (%)	85,506 (51.6)	59,785 (54.1)	25,721 (46.5)	
Body mass index, kg/m^2^	23.5 ± 3.3	23.5 ± 3.3	23.7 ± 3.1	< 0.001
Low income, *n* (%)	31,365 (18.9)	21,210 (19.2)	10,155 (18.3)	< 0.001
Smoking status				
Current or past smoker, *n* (%)	24,294 (14.7)	17,239 (15.6)	7,055 (12.7)	< 0.001
Alcohol consumption				
Heavy drinker, *n* (%)	8,402 (5.1)	5,579 (5.1)	2,823 (5.1)	0.657
Comorbidities				
Hypertension, *n* (%)	64,250 (38.7)	42,287 (38.3)	21,963 (39.7)	< 0.001
Dyslipidemia, *n* (%)	50,422 (30.4)	32,984 (29.9)	17,438 (31.5)	< 0.001
Diabetes mellitus, *n* (%)	22,762 (13.7)	14,623 (13.2)	8,139 (14.7)	< 0.001
Chronic kidney disease, *n* (%)	10,948 (6.6)	7,333 (6.6)	3,615 (6.5)	0.426
Asthma or COPD, *n* (%)	7,885 (4.8)	4,871 (4.4)	3,014 (5.4)	< 0.001
Cancer, *n* (%)	57,243 (34.5)	38,630 (35.0)	18,613 (33.6)	< 0.001
Amounts of exercise				
≥ 500 MET-min/week, *n* (%)	80,102 (48.3)	35,871 (32.5)	44,231 (79.9)	< 0.001

COPD, chronic obstructive pulmonary disease; MET, metabolic equivalent of task.

In terms of comorbidities, physically active individuals showed a higher proportion of hypertension (39.7% vs. 38.3%, *p* < 0.001), dyslipidemia (31.5% vs. 29.9%, *p* < 0.001), diabetes mellitus (14.7% vs. 13.2%, *p* < 0.001), and asthma or COPD (5.4% vs. 4.4%, *p* < 0.001) than those who were inactive. Physically active individuals engaged in significantly more exercise compared to those who were inactive (79.9% vs. 32.5% for ≥ 500 MET-min/week, *p* < 0.001). However, no significant differences were observed in alcohol consumption and chronic kidney disease between the two groups (*p* > 0.05 for both).

### Impact of exercise on mortality

**Figure 1:**
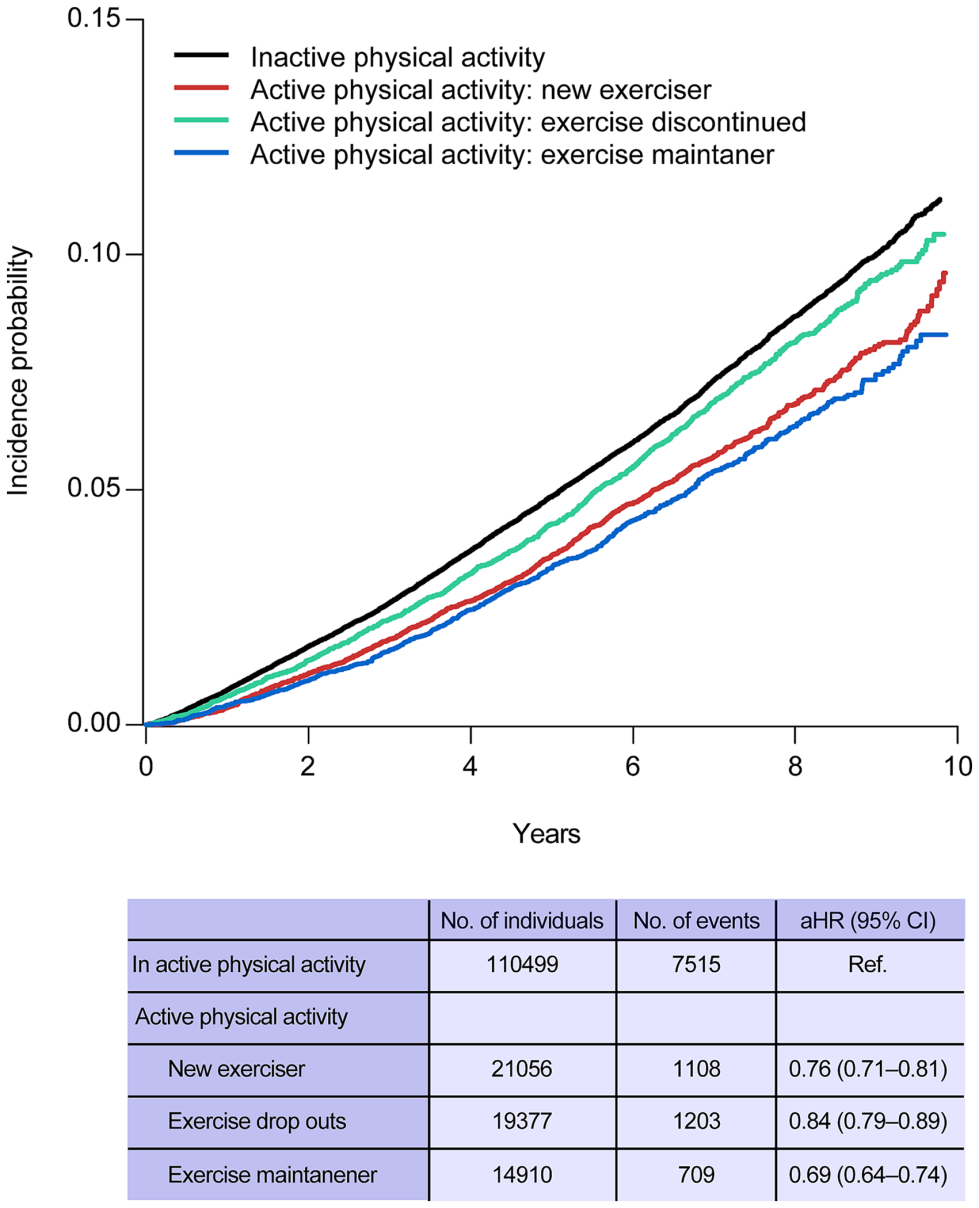
Kaplan–Meier curves of incidence probability of mortality according to the physical activity status. aHR, adjusted hazard ratio; CI, confidence interval.

During a mean follow-up of 6.2 ± 2.1 years, 10,535 (6.4%) individuals with bronchiectasis died. As shown in Figure 1, the individuals with bronchiectasis who engaged in active physical activity exhibited lower mortality rates than those who engaged in inactive physical activity. The risk reduction of mortality was highest in the exercise maintainers group (adjusted hazard ratio [aHR] = 0.69, 95% CI = 0.64–0.74) and lowest in the exercise discontinued group (aHR = 0.84, 95% CI = 0.79–0.89).

Regarding the amount of exercise and risk of mortality, the greatest reduction in mortality was observed in individuals who performed exercise ≥1,000 MET-min/week (aHR = 0.73, 95% confidence interval [CI] = 0.70–0.77), followed by 500–999 MET-min/week (aHR = 0.74, 95% CI = 0.68–0.81) and < 500 MET-min/week (aHR = 0.89, 95% CI = 0.83–0.96) (Table 2).

**Table 2:** Effect of physical activity on the risk of mortality.

		No. of individuals	Total		
			No. of events	IR (/1,000 PY)	aHR (95% CI)
Overall	Inactive physical activity	110,499	7,515	10.8	1 (Reference)
	Active physical activity				
	<500 MET-min/week	11,112	750	10.7	0.89 (0.83–0.96)
	500–999 MET-min/week	11,407	589	8.1	0.74 (0.68–0.81)
	≥1,000 MET-min/week	32,824	1,681	8.2	0.73 (0.70–0.77)
	*p*-value				< 0.001
New exerciser	Inactive physical activity	110,499	7,515	10.8	1 (Reference)
	Active physical activity				
	<500 MET-min/week	457	13	4.4	0.50 (0.29–0.85)
	500–999 MET-min/week	2,384	99	6.4	0.71 (0.58–0.86)
	≥1,000 MET-min/week	18,215	996	8.7	0.77 (0.72–0.82)
	*p*-value				< 0.001
Exercise discontinued	Inactive physical activity	110,499	7,515	10.8	1 (Reference)
	Active physical activity				
	<500 MET-min/week	10,377	723	11.0	0.91 (0.84–0.98)
	500–999 MET-min/week	7,520	418	8.9	0.75 (0.68–0.83)
	≥1,000 MET-min/week	1,480	62	6.9	0.77 (0.60–0.99)
	*p*-value				< 0.001
Exercise maintainers	Inactive physical activity	110,499	7,515	10.8	1 (Reference)
	Active physical activity				
	<500 MET-min/week	278	14	7.6	0.86 (0.51–1.45)
	500–999 MET-min/week	1,503	72	7.5	0.74 (0.58–0.93)
	≥1,000 MET-min/week	13,129	623	7.7	0.68 (0.63–0.74)
	*p*-value				< 0.001

IR, incidence rate; aHR, adjusted hazard; CI, confidence interval; MET, metabolic equivalent of task.

## Discussion

The significant findings of this study are as follows. First, approximately two-third of individuals with bronchiectasis did not engage in active physical activity. Second, mortality significantly decreased in those who engaged in active physical activity. Third, the reduction in mortality was highest among individuals in the exercise maintainers group and the lowest among exercise discontinued. Fourth, although the amount of active physical activity correlates with the risk reduction of mortality, even a small amount of physical activity can reduce the risk of mortality in individuals with bronchiectasis.

The detrimental effects of physical inactivity are well-documented; however, a significant proportion of individuals continue to fall short of meeting recommended physical activity levels. Recent data indicate that nearly one-third of adults globally, approximately 1.8 billion people, remain physically inactive (45). In patients with bronchiectasis, the prevalence of physical inactivity varies globally, ranging from 40.9 to 73.7% (Table 3) (46–49). Notably, our findings indicate that the proportion of physically inactive individuals with bronchiectasis in Korea is considerably high, far exceeding half of those with bronchiectasis. Recently, physical inactivity has been a significant concern in the Asia-Pacific region, driven by factors such as aging populations, climate change, insufficient infrastructure, and the lingering impacts of the coronavirus 2019 pandemic (50). Considering these circumstances, it is time to focus on the significance of physical inactivity in patients with bronchiectasis, particularly in the Asia-Pacific region.

**Table 3:** Summary of studies assessing physical inactivity prevalence in bronchiectasis.

Study	Country	Data source	Measurement of physical inactivity	Definition of physical inactivity	Prevalence of physical inactivity
Alcaraz-Serrano et al. (46)	Spain	A tertiary care hospital	A tri-axial accelerometer (SenseWear Armband; BodyMedia Inc., Pittsburgh, PA, United States)	< 6,290 steps per day	26/53 (49.1%)
Bradley et al. (47)	UK	Respiratory clinics at the three selected hospital sites	ActiGraph GT3X+ accelerometer	< 5,000 steps per day	23/55 (41.8%)
de Camargo et al. (48)	Brazil	A tertiary university hospital	Pedometer (Yamax Power Walker, model PW-610; Yamax Corp, Tokyo, Japan)	< 7,500 steps per day	61/149 (40.9%)
Pehlivan et al. (49)	Turkey	Pulmonology outpatient clinics	International Physical Activity Questionnaire (IPAQ) Short Form	Did not meet any of the following criteria: (a) engaging in vigorous activity ≥20 min/day on 3 or more days/week, (b) engaging in moderate-intensity activity or walking for ≥30 min/day on 5 or more days per week, OR (c) achieving a minimum of 600 MET-minutes per week through a combination of walking, moderate-intensity, or vigorous-intensity activities on 5 or more days per week.	14/19 (73.7%)

IPAQ, International Physical Activity Questionnaire; MET, Metabolic equivalent of task.

Our findings indicate that active physical activity can effectively decrease mortality risk in individuals with bronchiectasis. Two interesting findings were observed when we assessed physical activity in terms of maintenance and amount. First, although the maintenance of active physical activity showed the highest reduction in mortality, mortality reduction was observed even in individuals in the exercise discontinued group. Second, although there is a correlation between the amount of physical activity and mortality reduction, even individuals who engaged in physical activity below the recommended threshold (approximately more than 600 MET-min/week) showed a significant decrease in mortality (51). Therefore, our study indicates that it is important to encourage individuals with bronchiectasis to maintain physical activity regardless of the amount of exercise because this can yield substantial long-term survival benefits.

This study has several limitations that warrant consideration. First, certain factors not assessed in this study may influence the outcomes. Key variables impacting mortality, such as disease severity—including exacerbation history, lung function, specific treatments, and bacterial colonization—could not be analyzed due to the lack of available data (52–54). Future research should incorporate these variables to understand better the relationship between physical activity and mortality in individuals with bronchiectasis. Second, although we showed that the amount of physical activity is associated with reduced mortality in patients with bronchiectasis, this measure was indirectly assessed. Furthermore, since we used self-reported data, there might be a recall bias. Thus, future studies that directly measure the intensity and duration of exercise, such as using accelerometers, are needed to provide a more comprehensive understanding of the impact of physical activity on the mortality in individuals with bronchiectasis. Third, as this study was conducted in a single Asian country, the generalizability of the results to populations in other regions may be limited.

In conclusion, actively engaging in physical activity is associated with a decreased mortality risk in individuals with bronchiectasis. Therefore, more active physical activity is recommended for this population.

## Data Availability

The data analyzed in this study is subject to the following licenses/restrictions: the data that support the findings of this study are available from Korea NHIS but restrictions apply to the availability of these data, which were used under license for the current study, and so are not publicly available. Requests to access these datasets should be directed to https://nhiss.nhis.or.kr/bd/ab/bdaba001cv.do.
